# A High Grain Diet Dynamically Shifted the Composition of Mucosa-Associated Microbiota and Induced Mucosal Injuries in the Colon of Sheep

**DOI:** 10.3389/fmicb.2017.02080

**Published:** 2017-10-26

**Authors:** Yue Wang, Lei Xu, Junhua Liu, Weiyun Zhu, Shengyong Mao

**Affiliations:** Jiangsu Key Laboratory of Gastrointestinal Nutrition and Animal Health, Laboratory of Gastrointestinal Microbiology, College of Animal Science and Technology, Nanjing Agricultural University, Nanjing, China

**Keywords:** colon, microbiota, high grain diet, mucosal injury, sheep

## Abstract

This study investigated the dynamic shifts in mucosa-associated microbiota composition and mucosal morphology in the colon of sheep fed a high grain (HG) diet. A total of 20 male sheep were randomly assigned to four groups (*n* = 5 for each). The sheep in first group received hay diet. The animals in other 3 groups were fed an HG diet for 7 (HG7), 14 (HG14), or 28 (HG28) days, respectively. Colonic digesta samples were collected to determine the pH and the concentrations of volatile fatty acid (VFA) and lactate. The colonic mucosa was sampled to characterize the bacterial communities using Illumina MiSeq sequencing and to determine mRNA expression levels of cytokines and tight junction protein genes using quantitative real-time PCR. As time advanced, results revealed that colonic pH linearly decreased (*P* = 0.007), and the concentrations of total VFA linearly increased (*P* < 0.001). Microbial analysis showed that an HG diet linearly reduced (*P* < 0.050) the diversity and richness of the colonic microbiota. The principal coordinate analysis results showed that the colonic mucosa-associated bacterial communities of the four groups significantly shifted with number of days fed an HG diet. At the genus level, HG feeding significantly increased the relative abundance of some taxa including *Prevotella*, *Coprococcus*, *Roseburia*, and *Clostridium_sensu_stricto_1*, and decreased the proportion of *Treponema,* and the percentage of these taxa was not affected by days fed an HG diet. The microscopic examination showed that HG feeding caused the mucosal epithelial injury. The RT-PCR results showed that the mRNA expression of claudin-1 (*P* = 0.038), IL-1β (*P* = 0.045), IL-6 (*P* = 0.050), and TNF-α (*P* = 0.020) increased linearly with number of days fed an HG diet. The correlation analysis revealed significant correlation between the colonic mucosal mRNA expression of cytokines and mucosal bacterial composition. Generally, HG feeding increased colonic fermentation and altered colonic mucosal bacterial communities, which eventually caused colonic mucosal damage and led to colonic dysfunction, and these changes occurred gradually over at least 4 weeks.

## Introduction

In modern ruminant production systems, feeding high grain (HG) diets to high producing ruminant animals has become a common strategy for increasing milk yields and maximizing economic efficiency (Krause and Oetzel, 2005; Tufarelli and Laudadio, 2011; Gao and Oba, 2014). However, this feeding strategy poses a challenge for the epithelium and microbiota of the gastrointestinal (GI) tracts in these animals (Chen et al., 2011; Liu et al., 2013). It has been reported that HG diet feeding had adverse impacts on rumen function and health, such as rumen acidosis and ruminitis (Klevenhusen et al., 2013). Previous studies reported that ruminants can adapt to HG diets which required at least 3 weeks for the ruminal microbial adaptation (Tajima et al., 2001) as well as morphological and functional adaptation of ruminal epithelium (Bannink et al., 2008; Etschmann et al., 2009; Górka et al., 2017).

Feeding diets containing high levels of a readily fermentable carbohydrate also increased the microbial fermentation in the hindgut, which may be due to the increase in the amount of starch flowing into the hindgut from rumen and small intestine (Owens et al., 1986; Gressley et al., 2011; Li et al., 2012), and the characteristic of high concentrations of volatile fatty acids (VFA) in the digesta (Liu et al., 2014; Ye et al., 2016). However, the buffering capacity of the hindgut is not as strong as that of the rumen, due to the absence of saliva and protozoa (Erdman, 1988; Hume, 1997). Therefore, the hindgut has no natural defense against acidosis and VFA toxicity, which may lead to an unhealthy GI environment, eventually leading to dramatic alterations in microbial compositions.

It is known that the colonic epithelium represents a physical and immunological barrier. Increased hindgut fermentation can result in a decline in the pH of the large intestinal, an increase in endotoxin concentration in the digesta, and damage to the gut epithelium (Liu et al., 2014; Ye et al., 2016). Previous studies have found that massive hindgut fermentation led to inflammation and laminitis, which may be induced by amines, endotoxins or bacteria that are able to across through a breach in the intestinal barrier (Gozho et al., 2006; Crawford et al., 2007).

Although many studies to date have assessed the adaptation of the bacteria associated with ruminal epithelium in response to dietary changes in cows (Tajima et al., 2001; Chen et al., 2011; Petri et al., 2013b) and goats (Liu et al., 2015), as well as the morphological and functional (Etschmann et al., 2009; Steele et al., 2009, 2011) adaptation of the ruminal epithelium in ruminants, little information is available on the time course of the mucosal microbial communities and epithelial functional adaption of hindgut to an HG diet, despite the fact that the hindgut of a ruminant also plays an important role in the animal’s overall health, productivity and welfare (Gressley et al., 2011).

Here, we hypothesized that there is a time course of adaptation of colonic mucosa-associated microbiota composition to an HG diet. We also hypothesized that feeding an HG diet may result in excessive colonic fermentation and a dynamic shift in the composition of colonic mucosal bacterial communities, which may contribute to local inflammatory responses in the colon of sheep. Therefore, this study aimed to characterize the time course of mucosa-associated microbia and mucosal morphological adaptation in response to an abrupt increase in the proportion of grain in the diet for sheep.

## Materials and Methods

### Animals and Sampling

All of the experiments were discussed and approved by Nanjing Agricultural University, Jiangsu province, China, according to the Animal Care and Use Committee of Nanjing Agricultural University. The protocol was approved by The State Science and Technology Commission of China.

This animal experiment was carried out at the experimental station of Nanjing Agricultural University in Jiangsu Province of China during the period from October 20th, 2015 to December 19th, 2015. In order to minimize individual differences caused by sex, a total of 20 male sheep (Hu Sheep, aged 180 days, with the average weight of 25.60 ± 0.41 kg) were used in the current study. All animals were randomly assigned to four groups, each containing five animals, and were kept in individual cages (1.2 × 1.4 m). During the 4-week preparation period, all sheep were fed a forage-based diet containing 96.4% hay and 3.6% mineral and vitamin premix (Supplementary Table S1) *ad libitum*, and had free access to clean water, in order to establish a similar colonic microbial community composition. In the adaptation stage following the preparation period, three HG groups were fed a step-up diet (the HG was increased by 15 × 3.50% of body weight per day) for 4 days, until they were fed with a 60% concentrate mix (Supplementary Table S1); the control (CON) group continued to receive the forage-based diet. Following adaptation, the CON group were fed the forage-based diet for an additional 28 days. In contrast, the HG groups received an HG diet that continued for 7 (HG7), 14 (HG14), and 28 (HG28) days, respectively. The animals were fed with equal portions twice-daily, at 0830 and 1630. The body weight of the sheep was measured on the first day of every week before feeding, and their health was continually monitored.

At the end of each feeding period, the sheep were slaughtered at 4–5 h after their last feeding for sampling. Sheep in the CON group were slaughtered after 28 days of hay feeding, and sheep in the HG7, HG14, and HG28 groups were slaughtered after 7, 14, and 28 days since the shift to HG feeding, respectively. Colonic digesta samples were collected and pH values were assessed immediately. The first part of each colonic digesta sample was diluted by mixing with a double amount of distilled water; the mixture was then centrifuged at 2,000 × *g* for 10 min to separate the solid residues and liquid. Supernatant liquid samples were kept at -20°C for further VFA (Qin, 1982) and lactate concentration detection (Barker and Summerson, 1941). The second part of the digesta samples was placed in a 65°C oven for 48 h then shattered until the diameter of the samples was below 1 mm. After that, the shattered samples were stored at -20°C until the analysis of the starch content (Liu et al., 2014). The colonic mucosal samples were scraped using sterile slides, and then stored at -80°C for subsequent DNA extraction. Intact colonic tissue samples were collected and washed with ice-cold phosphate-buffered saline, and then divided into two parts. One part of samples was cut into 0.4 × 0.4 cm sections, then kept at -80°C for subsequent RNA extraction, the other part of samples was fixed in 4% paraformaldehyde (Sigma, St. Louis, MO, United States) for histomorphometric microscopy analysis.

### Physiological Parameters Measurements

Colonic pH value was detected by a pH-meter (HI 9024C, HANNA Instruments, Woonsocket, RI, United States). The levels of VFA were detected through the GC method (capillary column: 30 m × 0.32 mm × 0.25 mm film thickness; column temperature = 110°C; injector temperature = 180°C; detector temperature = 180°C) (Qin, 1982). Lactate levels was determined using the Barker method (Barker and Summerson, 1941). The starch content in the colonic digesta was determined using a Megazyme Total Starch Assay Kit (Megazyme International Ireland Limited, Wicklow, Ireland). In brief, 100 mg of milled digesta samples and 2 mL of dimethyl sulphoxide (DMSO) were added to a glass test tube and mixed fully to promote starch gelatinisation. 3 mL of thermostable a-amylase was added to the tube and incubated in a boiling water bath to hydrolyze starch to glucose. Six minutes later, added 0.1 mL of amyloglucosidase and incubated at 50°C for 30 min to promote starch hydrolysis completely. The absorbance of each sample and blank was measured at 510 nm using MultiSkan Go spectrophotometer (Thermo Scientific, Finland).

### Bacterial DNA Extraction of Colonic Mucosa

A total of 0.25 g of colonic mucosa was used for DNA extraction. The process was conducted using a QIAamp Fast DNA Stool Mini Kit (Qiagen, Hilden, Germany) and then quantified by a Nanodrop 2000 spectrophotometer (Thermo Fisher Scientific, Waltham, MA, United States). The mucosal sample, 1 mL of Inhibit EX buffer, and 100 mg of zirconium beads (0.1 mm) were placed in a centrifuge tube respectively and homogenized by Mini-Beadbeater-1 (Biospec Products Inc.) to improve lysis efficiency. The mixture was heated at 95°C for 5 min to increase the production of DNA. After that, the mixture was centrifuged for 1 min, and the obtained supernatant fluid was mixed with 15 μL proteinase K in a new microcentrifuge tube to purify the DNA. The DNA samples were kept at -80°C for further analysis.

### PCR Amplification and Illumina MiSeq Sequencing

The primers used for the V3–V4 region of the bacteria 16S ribosomal RNA (16S rRNA) gene amplification were 338F (5′-barcode- ACTCCTRCGGGAGGCAGCAG-3’) and 806R (5′-GGACTACCVGGGTATCTAAT-3′), with an amplicon length of approximately 470 bp. The amplification process included initial denaturation at 95°C for 2 min, 25 cycles of denaturation at 95°C for 30 s, annealing at 55°C for 30 s, elongation at 72°C for 30 s, and final extension at 72°C for 5 min. The PCR products were electrophoresed on 2% agarose gels. After that, the purification was performed using an AxyPrep DNA Gel Extraction Kit (Axygen Biosciences, Union City, CA, United States). The quantification of amplicons was carried out utilizing QuantiFluor^TM^ -ST (Promega, United States). The Amplicons from all samples were used to construct a sequencing bank with an Illumina TruSeq DNA Sample Preparation Kit (Illumina, San Diego, CA, United States). An Illumina TruSeq PE Cluster and Synthesis (SBS) Kit was applied to perform cluster generation, template hybridization, isothermal amplification, linearization, blocking and denaturization, and hybridization of the sequencing primers. Paired-end sequencing 2 × 250 base pairs (bp) was carried out to sequence all libraries on an Illumina MiSeq forum in accordance with standard methods (Caporaso et al., 2012).

The data were processed using the QIIME (version 1.70) software package (Campbell et al., 2010). According to 6 bp sample-specific barcodes, the 16S rRNA reads were decoded and high quality sequences were selected. The Quality filters used to select sequences were a length greater than 250 bp, the absence of vague base ‘N,’ and an average base quality score of higher than 25. On the basis of a sequence similarity level of 97%, sequences were clustered into operational taxonomic units (OTUs) using UPARSE (Edgar, 2010). Within each OTU, the representative sequences defined for the most abundant sequences were identified, and then individually assigned to the core set of Greengenes 13.5 (Campbell et al., 2010). Taxonomy assignment was achieved by the Bayesian classifier of the Ribosomal Database Project (Wang et al., 2007), via the contrast between representative sequences in every OTU cluster and the Greengenes database (DeSantis et al., 2006), using PyNAST (Caporaso et al., 2010) with the default parameters set by QIIME. Alpha diversity such as the abundance-based coverage estimator (ACE), the Chao1 value, the Shannon index, and the simpson index were evaluated using the QIIME (version 1.70) software package. A cladogram of the representative sequences was set up using FastTree (Price et al., 2009). Unweighted Unifrac distance-based principal coordinate analysis (PCoA), together with a molecular variance analysis (AMOVA), were carried out to evaluate the dissimilarities amongst the specimens, via version 1.29 of the MOTHUR program (Schloss et al., 2009).

The 16S sequencing data obtained from the samples that were analyzed in the present study were submitted to the Sequence Read Archive (SRA) under accession SRP 114490.

### Mucosal RNA Extraction of Colon and Real-Time PCR

The extraction of RNA from the colonic mucosa was performed using TRIzol (Takara Bio, Otsu, Japan), based on a previously used method (Chomczynski and Sacchi, 1987). The quantification of RNA concentration was evaluated utilizing a NanoDrop spectrophotometer (Thermo Scientific Inc., Wilmington, DE, United States). A 1.4% agarose-formaldehyde gel electrophoresis was used to verify the quality of the RNA samples. The concentration of each RNA sample was then adjusted to 500 ng/μl and stored at -80°C. One microgram of total RNA was reverse-transcribed using a PrimeScript^®^ RT reagent Kit with gDNA Eraser (Takara Bio, Otsu, Japan).

All primers used in the present study were showed in Supplementary Table S2. The primers for inflammatory cytokines (IL-1β, IL-6, IL-10, TNF-α and IFN-γ) and tight junction proteins (claudin-1, claudin-4, claudin-7, occludin, and ZO-1) were designed by Liu et al. (2013). The primers for toll-like receptor (TLR-3 and TLR-4) were designed by Liu et al. (2015). Primers of GAPDH were designed by Wang et al. (2009). All primers were synthesized by Invitrogen Life Technologies (Invitrogen, Shanghai, China). Target gene expression and GAPDH were quantified by the ABI 7300 real-time PCR system (Applied Biosystems, Foster, City, CA, United States), using fluorescence detection of SYBR^®^ Green dye. The amplification program was described by Ye et al. (2016). Every sample comprised 1–10 ng cDNA in a 2 x SYBR^®^ Green PCR master mix (Takara Bio, Otsu, Japan), as well as each primer, in a volume of 20 μl. All samples were analyzed in triplicate on one plate per gene. The mRNA abundance of target genes was normalized using the house-keeping gene GAPDH and calculated using the 2^-Δ^ ^ΔC_T_^ method.

### Statistical Analysis

The bacterial prevalence obtained, and the mRNA expression of genes associated with inflammatory cytokines and tight junction proteins, was analyzed using one-way ANOVA procedures (SPSS version 22.0, SPSS, Inc.). The hypotheses were tested using preplanned orthogonal (treatment: CON vs. HG) and polynomial contrasts (linear and quadratic effects), accounting for the unequal durations of time that sheep were fed the HG diet. Polynomial contrasts included the CON treatment, and statistical significance was defined as *P* < 0.05.

The non-parametric Kruskal–Wallis *post hoc* test was carried out to calculate the microbial data (SPSS version 22.0, SPSS, Inc.), and the *P*-values obtained were corrected using a false discovery rate (FDR) according to the Benjamini–Hochberg method (Benjamini and Hochberg, 2000). FDR-corrected *P*-values of below 0.05 (FDR < 0.05) were defined as significance. Correlative relationships between colonic inflammatory cytokine expression and mucosal-associated microbiota in the colon were assessed using Spearman correlation test, via GraphPad Prism version 6.00 (GraphPad Software, San Diego, CA, United States).

## Results

### pH and Concentrations of VFA in the Colonic Digesta of Sheep

As shown in **Table 1**, colonic pH decreased from 6.70 for CON group to 6.55, 6.52, and 6.11 for HG7, HG14, and HG28 groups (linear, *P* = 0.007), respectively. The total VFA concentration (slaughter point) increased linearly from 27.12 μmol/g for CON group to 43.68 for HG7 group, 48.56 μmol/g for HG14 group, and 65.49 μmol/g for HG28 group (*P* < 0.001). Acetate, propionate, and butyrate levels increased linearly with number of days fed an HG diet (*P* < 0.001, for all), and the lactate concentration also increased linearly with number of days fed an HG diet (*P* = 0.015). The concentration of valerate decreased from CON group to HG7 group, and then recovered to the level of CON group (quadratic, *P* < 0.001). The concentrations of starch and isobutyrate increased from CON group to HG7 group and then decreased at HG14 group, followed by an increase at HG28 group (cubic, *P* = 0.030, and *P* < 0.001, respectively). The level of isovalerate (*P* > 0.05) was not affected by days fed HG diet.

**Table 1:** Colonic pH and concentrations of volatile fatty acid (VFA) in colonic digesta of sheep fed hay (CON) and high grain diets (HG 7–28)^a^.

Items	CON	HG7	HG14	HG28	SEM	*P*-value			
						Con vs. HG	Linear	Quadratic	Cubic
pH	6.70	6.55	6.52	6.11	0.080	<0.001	0.007	0.339	0.462
TVFA μmol/g	27.12	43.68	48.56	65.49	3.343	<0.001	<0.001	0.944	0.069
Acetate μmol/g	21.48	35.97	38.37	51.59	2.645	<0.001	<0.001	0.781	0.041
Propionate μmol/g	3.70	3.64	4.50	6.60	0.354	<0.001	<0.001	0.049	0.902
Isobutyrate μmol/g	0.42	0.65	0.33	0.61	0.033	<0.001	0.154	0.597	<0.001
Butyrate μmol/g	1.03	3.17	5.02	6.27	0.507	<0.001	<0.001	0.395	0.903
Isovalerate μmol/g	0.24	0.22	0.16	0.18	0.018	<0.001	0.111	0.600	0.496
Valerate μmol/g	0.25	0.03	0.17	0.28	0.024	<0.001	0.022	<0.001	0.001
Starch content %	0.48	2.47	0.53	1.29	0.003	0.006	0.851	0.325	0.030
Lactate μmol/g	2.30	2.39	3.02	3.45	0.190	<0.001	0.015	0.633	0.657

^a^*Sheep assigned to CON (*n* = 5), HG7 (*n* = 5), HG14 (*n* = 5), and HG28 (*n* = 5) received a high grain diet for 0, 7, 14, and 28 days, respectively*.

### The Temporal Frame of Dynamic Shifts in the Diversity and Richness of Colonic Mucosa-Associated Bacterial Microbiota

As shown in Supplementary Figure S1, the rarefaction curves approached a plateau, suggesting that sequencing depth was sufficient to detect the vast majority of bacteria present. The number of OTUs, ACE, Chao 1, and the Shannon index of the mucosa-associated microbial communities are shown in **Table 2**. At the 97% similarity level, the OTU numbers, ACE value, and Chao 1 decreased linearly with number of days fed an HG diet (*P* < 0.001). However, the Shannon index was only affected by HG treatment (*P* < 0.001), while the time of being fed an HG diet had no effect on the indices (*P* > 0.05).

**Table 2:** Effects of feeding a high-grain (HG) diet on the average richness and diversity of colonic mucosa-associated bacteria community at the 3% dissimilarity level.

Items	CON	HG7	HG14	HG28	SEM	*P*-value		
						Treatment	Linear	Quadratic
OTU^a^ numbers	1012	488	536	638	53	<0.001	<0.001	<0.001
ACE^b^	1350	652	676	855	70	<0.001	<0.001	<0.001
Chao1	1313	650	680	830	68	<0.001	<0.001	<0.001
Shannon	3.74	4.19	4.09	4.35	0.101	<0.001	0.061	0.620

^a^*OTU, operational taxonomic units. ^b^ACE, abundance-based coverage estimator*.

**FIGURE 1:**
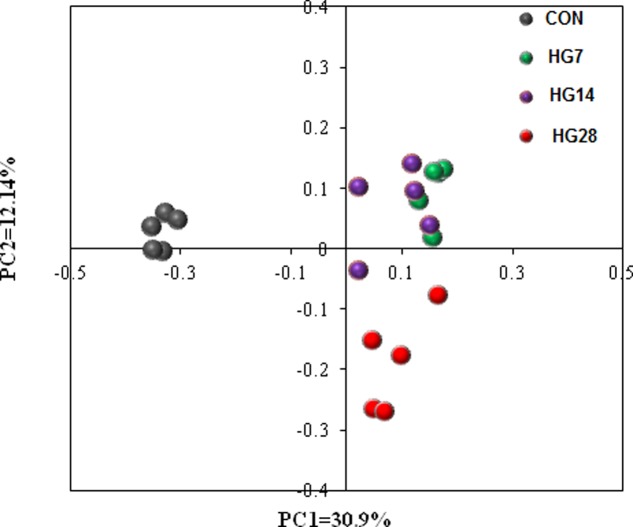
Principal coordinate analysis (PCoA) of bacterial communities in colonic mucosa of sheep fed hay (CON) and a high grain (HG7–28) diet.

The results of unweighted UniFrac distance-based PCoA demonstrated that the mucosa-associated bacterial communities of CON, HG7, HG14, and HG28 groups were independently gathered (**Figure 1**). The results of unweighted distance-based analysis of AMOVA also showed significant dissimilarities among four groups (*F*s = 4.984, *P* < 0.001, among CON, HG7, HG14 and HG28 groups; *F*s = 8.972, *P* < 0.001, CON vs. HG7; *F*s = 7.570, *P* < 0.001, CON vs. HG14; *F*s = 6.784, *P* < 0.001, CON vs. HG28; *F*s = 2.534, *P* < 0.001, HG7 vs. HG14; *F*s = 2.930, *P* < 0.001, HG7 vs. HG28; *F*s = 2.524, *P* < 0.001, HG14 vs. HG28).

In order to understand how microbial communities adapt to an HG diet, the number of OTUs shared among the four groups was evaluated (Supplementary Figure S2). Of the 2,316 OTUs identified in colonic mucosa, 461 OTUs were common among all the bacterial communities of the four groups; 714 (8.40% of the total sequences) in CON, 51 (0.17%) in HG7, 81 (0.26%) in HG14, and 242 (1.12%) in HG28 were unique.

### The Temporal Dynamic Shifts in the Composition of Colonic Mucosa-Associated Bacterial Microbiota

In the current study, a total of 712,120 qualified 16S rRNA gene sequences were detected from 20 samples, with average sequence number of 35,606 ± 3490 per sample. Total sequences were assigned to 21 phyla; the three most phyla in the CON group were Firmicutes, Spirochaetae, and Proteobacteria, whereas the three most common phyla in the HG groups were Firmicutes, Bacteroidetes, and Proteobacteria (Supplementary Figure S3). As shown in **Table 3**, HG feeding significantly affected the abundance of seven phyla. Of them, as compared with the CON group, the abundance of Firmicutes (FDR = 0.022), Bacteroidetes (FDR = 0.022), and Actinobacteria (FDR = 0.022) were higher in the HG groups, while the abundance of Spirochaetae (FDR = 0.022) and Elusimicrobia (FDR = 0.022) were lower in the HG groups, and the percentage of these seven phyla was not affected by days fed an HG diet. The abundance of Cyanobacteria (FDR = 0.022) and Candidate_division_TM7 (FDR = 0.025) decreased from CON group to HG14 group and then recovered to the CON level.

**Table 3:** Effects of feeding a high-grain (HG) diet on average relative abundance of phylum (% of total sequences) in colonic mucosa.

Phylum	CON	HG7	HG14	HG28	SEM	FDR
Firmicutes	29.28^b^	58.22^a^	54.46^a^	68.68^a^	3.867	0.022
Bacteroidetes	8.44^b^	25.26^a^	21.17^a^	20.84^a^	1.735	0.022
Proteobacteria	13.68	14.00	22.00	6.50	2.396	0.185
Spirochaetae	46.36^a^	0.01^b^	0.71^ab^	0.16^b^	4.734	0.022
Actinobacteria	0.29^b^	1.81^a^	0.75^ab^	2.28^a^	0.286	0.022
Verrucomicrobia	0.70	0.39	0.29	0.52	0.087	0.404
Tenericutes	0.19	0.11	0.38	0.34	0.050	0.190
Cyanobacteria	0.54^a^	0.02^c^	0.03^bc^	0.29^ab^	0.058	0.022
Lentisphaerae	0.19	<0.01	0.02	0.01	0.025	0.061
Unclassified bacteria	0.05	0.10	0.11	0.17	0.028	0.611
Fibrobacteres	0.09	<0.01	0.05	0.05	0.013	0.185
Elusimicrobia	0.12^a^	0.00^c^	0.00^bc^	0.03^ab^	0.014	0.022
Candidate_division_TM7	0.05^a^	0.01^b^	0.01^b^	0.10^a^	0.015	0.025
Deinococcus-Thermus	<0.01	0.05	0.01	0.01	0.007	0.123
Synergistetes	<0.01	<0.01	<0.01	0.01	0.001	0.364
Chloroflexi	<0.01	<0.01	<0.01	0.01	0.001	0.281
Fusobacteria	<0.01	<0.01	<0.01	<0.01	0.001	0.323
Acidobacteria	<0.01	<0.01	<0.01	<0.01	0.001	0.052
Deferribacteres	<0.01	<0.01	<0.01	<0.01	<0.001	0.454
Candidate_division_SR1	<0.01	<0.01	<0.01	<0.01	<0.001	0.604
BD1-5	<0.01	<0.01	<0.01	<0.01	<0.001	0.454

At the genus level, the predominant taxa (those with a relative abundance ≥ 1% in at least one treatment) are listed in Supplementary Figure S4. In total, 22 taxa were detected as the predominant taxa, and the taxa with a relative abundance greater than 10% in at least one group were *Treponema*, unclassified Ruminococcaceae, unclassified Lachnospiraceae, and *Helicobacter* (**Table 4**). As shown in **Table 4**, HG feeding significantly affected the abundance of 12 taxa. Of them, the abundance of unclassified Lachnospiraceae (FDR = 0.015), *Bacteroides* (FDR = 0.026), *Prevotella* (FDR = 0.026), *Roseburia* (FDR = 0.015), *Coprococcus* (FDR = 0.020), Clostridium_sensu_stricto_1 (FDR = 0.015), *Oscillibacter* (FDR = 0.015), *Bifidobacterium* (FDR = 0.017), and unclassified Erysipelotrichaceae (FDR = 0.015) significantly increased in the HG groups compared with the CON group, and the abundance of these phyla was not affected by days fed an HG diet. The abundance of *Treponema* (FDR = 0.015) decreased in the HG groups compared with the CON group, and duration of HG feeding had no significant effect on the abundance of these phyla. Dynamic shifts were detected in some taxa during the HG feeding period. The abundance of *Faecalibacterium* (FDR = 0.016) remained stable after HG feeding until an increase of the genus was observed in HG28. With an increasing duration of HG feeding, the abundance of *Helicobacter* (FDR = 0.017) decreased in HG7 group then recovered to the CON level in HG14 group, and then decreased in HG28 group.

**Table 4:** Effects of feeding a high-grain (HG) diet on average relative abundance of taxa (% of total sequences) in colonic mucosa.

Genus	CON	HG7	HG14	HG28	SEM	FDR
Unclassified Ruminococcaceae	17.35	21.33	22.29	19.17	1.407	0.527
Unclassified Lachnospiraceae	3.34^b^	19.67^a^	12.02^ab^	18.23^a^	1.823	0.015
*Halomonas*	2.78	9.01	6.07	3.24	0.890	0.058
*Bacteroides*	1.03^b^	8.39^a^	5.05^ab^	7.42^a^	1.159	0.026
*Prevotella*	0.18^b^	7.59^a^	8.05^a^	8.89^a^	1.607	0.026
*Alistipes*	0.98	4.53	2.07	1.62	0.457	0.261
*Campylobacter*	9.15	3.83	3.20	0.79	1.071	0.081
Unclassified Rikenellaceae	2.40	2.68	3.17	0.54	0.425	0.115
*Coprococcus*	0.23^b^	2.08^a^	3.65^a^	3.80^a^	0.612	0.020
*Roseburia*	0.09^b^	2.02^a^	1.11^ab^	4.59^a^	0.533	0.015
Unclassified Christensenellaceae	1.10	1.98	1.85	2.07	0.216	0.334
*Ruminococcus*	0.28	1.62	1.35	1.95	0.286	0.075
*Bifidobacterium*	0.05^b^	1.40^a^	0.37^ab^	1.73^a^	0.267	0.017
*Oscillibacter*	0.16^b^	1.11^ab^	3.13^a^	2.52^a^	0.367	0.015
Unclassified Prevotellaceae	1.43	1.11	0.45	0.42	0.182	0.115
*Blautia*	1.56	0.93	0.79	1.01	0.148	0.519
*Clostridium_sensu_stricto_1*	0.02^b^	0.80^a^	0.66^a^	2.86^a^	0.381	0.015
Unclassified vadinBB60	1.46	0.74	1.81	2.01	0.259	0.241
Unclassified Erysipelotrichaceae	0.09^b^	0.62^ab^	1.06^a^	1.16^a^	0.118	0.015
*Faecalibacterium*	0.01^b^	0.05^b^	0.04^b^	2.74^a^	0.414	0.016
*Treponema*	46.32^a^	0.01^b^	0.71^ab^	0.16^b^	4.730	0.015
*Helicobacter*	1.17^a^	0.00^b^	11.12^a^	0.01^b^	2.016	0.017

### Metagenomic Metabolic Functions of Colonic Mucosa-Associated Microbiota

**FIGURE 2:**
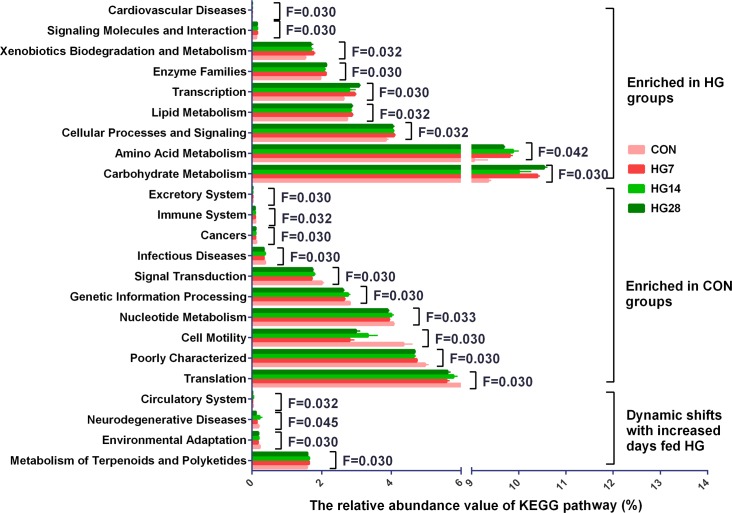
Influence of feeding a high grain (HG) diet on the abundance of KEGG pathways of colonic mucosal microbiota of sheep. Only the KEGG pathways that were significantly affected in percentage with increasing number of days of being fed an HG diet were presented. Significance was declared at FDR < 0.05.

In the 40 gene families detected in the colonic mucosal microbiomes (Supplementary Table S3), the majority of the genes that were obtained corresponded to membrane transport, carbohydrate metabolism, amino acid metabolism, replication and repair, translation, and energy metabolism. Of the 40 gene families, 24 were significantly differentially abundant among four groups (*P* < 0.05) (**Figure 2**). Compared with the CON group, HG-fed sheep had higher abundance of gene families carbohydrate metabolism (FDR = 0.030), amino acid metabolism (FDR = 0.042), cellular processes and signaling (FDR = 0.032), transcription (FDR = 0.030), lipid metabolism (FDR = 0.032), enzyme families (FDR = 0.030), xenobiotics biodegradation and metabolism (FDR = 0.032), signaling molecules and interaction (FDR = 0.030), and cardiovascular diseases (FDR = 0.030), and the abundance of these gene families was not affected by days fed an HG diet. In contrast, the abundance of 11 gene families, including translation (FDR = 0.030), poorly characterized (FDR = 0.030), cell motility (FDR = 0.030), nucleotide metabolism (FDR = 0.033), genetic information processing (FDR = 0.030), folding, sorting and degradation (FDR = 0.030), signal transduction (FDR = 0.030), infectious diseases (FDR = 0.030), cancers (FDR = 0.030), immune system (FDR = 0.032), and excretory system (FDR = 0.030), were lower compared with the CON group, and duration of HG feeding had no effect on the abundance of these gene families. The abundance of gene families neurodegenerative diseases (FDR = 0.045) and circulatory system (FDR = 0.032) did not change until a decrease was observed in HG28 group. The abundance of gene families environmental adaptation (FDR = 0.030) and metabolism of terpenoids and polyketides (FDR = 0.030) increased from CON group to HG7 group and then recovered to the CON level in the HG14 or HG28 group, respectively.

### Morphology of the Colonic Epithelium

**FIGURE 3:**
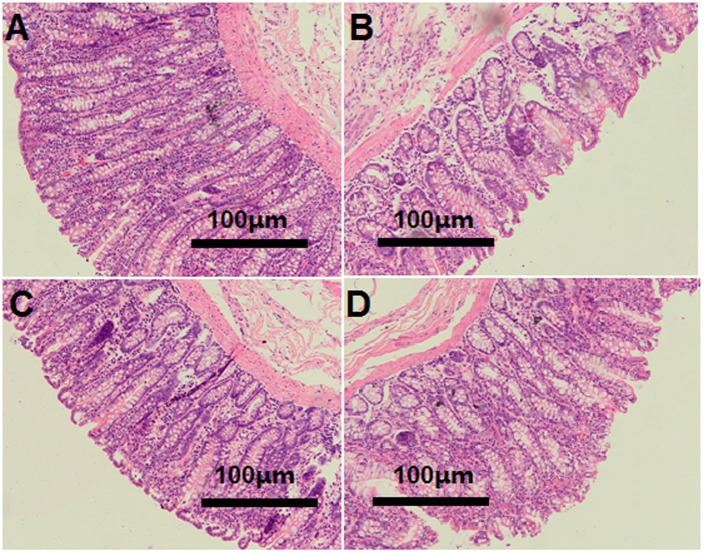
Histology of colon tissue, comparing hay-fed and a high grain (HG) diet-fed sheep. Light microscopy cross-section of colon tissue in the CON (**A**, scale bar = 100 μm), HG7 (**B**, scale bar = 100 μm), HG14 (**C**, scale bar = 100 μm), HG28 (**D**, scale bar = 100 μm).

The light microscopy cross-section results revealed that the inter-cryptal surface of the colonic epithelium in the CON group was partially covered by an irregular layer of mucus (**Figure 3A**). In contrast, colonic epithelium from the HG groups exhibited sloughing of the epithelial surface (**Figures 3B–D**).

### Relative mRNA Expression in the Colonic Tissue

**FIGURE 4:**
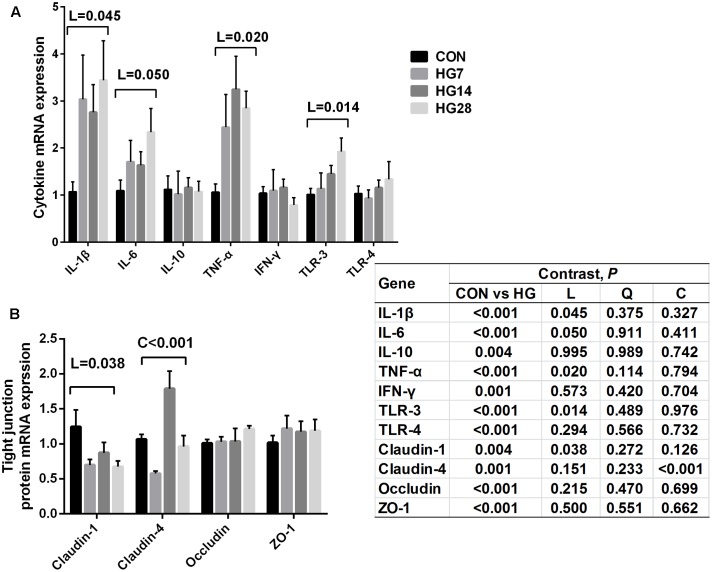
Effects of feeding a high grain (HG) diet on the relative mRNA expression of cytokines **(A)** and tight junction protein genes **(B)** in the colon of sheep. The amount of GAPDH mRNA levels was used to normalize the relative amount of each gene.

The relative gene expression of cytokines and tight junction proteins are shown in **Figure 4**. The mRNA expression of IL-1β (*P* = 0.045), IL-6 (*P* = 0.050), TNF-α (*P* = 0.020), and TLR-3 (*P* = 0.014) increased linearly with number of days fed an HG diet, whereas no significant differences were detected in the mRNA expression of IL-10, IFN-γ, and TLR-4 (*P* > 0.050) among four groups. An increase in the expression of claudin-1 (linear, *P* = 0.038) and claudin-4 (cubic, *P* = 0.009) was observed with number of days fed an HG diet.

### The Correlation between Colonic Inflammatory Cytokine Expression and Microbiota

**FIGURE 5:**
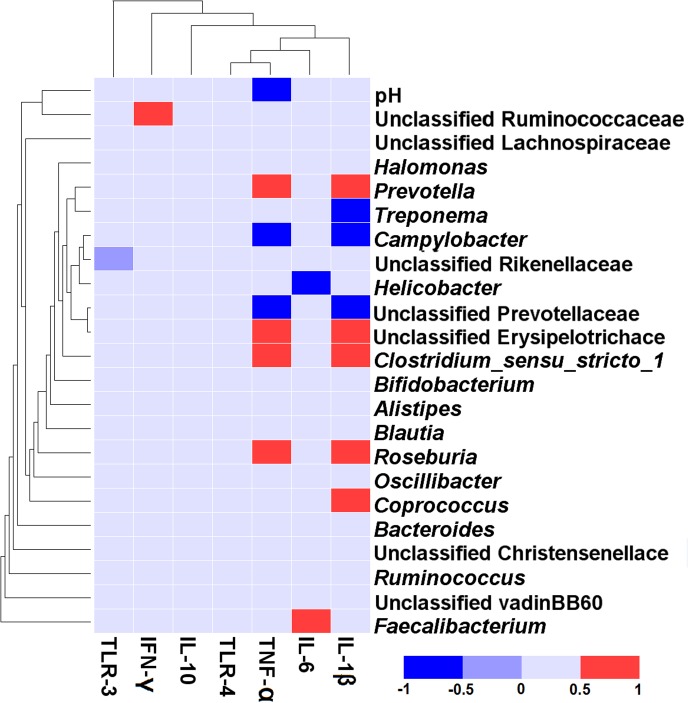
Correlation analysis between the abundance of taxa (at genus level), colonic pH, and the colonic inflammatory cytokine expression. Only the predominant genera (those with a relative abundance ≥ 1% in at least one treatment) in the colonic mucosa are shown. Cells are colored based on Spearman correlation coefficient. The red color represents a significant positive correlation (*P* < 0.05), the blue color represents a significant negative correlation (*P* < 0.05), and the light blue shows that the correlation was not significant (*P* > 0.05).

The correlative relationships between the abundant bacteria in colonic mucosa (relative abundance ≥ 1% in at least one treatment), colonic pH, and the colonic inflammatory cytokine expression are shown in **Figure 5**. Our results revealed a negative association between the mRNA expression of TNF-α and colonic pH (*r* = -0.517, *P* = 0.020), *Campylobacter* (*r* = -0.692, *P* < 0.001), and unclassified Prevotellaceae (*r* = –0.647, *P* = 0.002). In contrast, the level of TNF-α expression was positively correlated with *Prevotella* (*r* = 0.606, *P* = 0.005), *Clostridium_sensu_stricto_1* (r = 0.617, *P* = 0.004), unclassified Erysipelotrichace (*r* = 0.578, *P* = 0.008), and *Roseburia* (*r* = 0.588, *P* = 0.006). The mRNA expression of IL-1β was positively related to the abundance of five taxa [*Prevotella* (*r* = 0.659, *P* = 0.002), unclassified Erysipelotrichace (*r* = 0.592, *P* = 0.006), *Clostridium_sensu_stricto_1* (*r* = 0.672, *P =* 0.001), *Roseburia* (*r* = 0.632, *P* = 0.003), and *Coprococcus* (*r* = 0.605, *P* = 0.005)], and negatively related to the abundance of three taxa [*Treponema* (*r* = -0.535, *P* = 0.015), *Campylobacter* (*r* = -0.660, *P* = 0.002), and unclassified Prevotellaceae (*r* = -0.753, *P* = 0.001)]. The mRNA expression of IL-6 was positively correlated with the abundance of *Faecalibacterium* (*r* = 0.504, *P* = 0.024) and negatively correlated with the abundance of *Helicobacter*(*r* = -0.523, *P* = 0.018). The mRNA expression of IFN-γ was positively associated with the abundance of unclassified Ruminococcaceae (*r* = 0.614, *P* = 0.004).

## Discussion

### Temporal Dynamic Shifts in the Colonic Mucosa-Associated Bacterial Community during Adaptation to HG Diets

Previous studies have investigated the time course of ruminal microbial adaptation to HG diets in ruminants by PCR technology (Tajima et al., 2000, 2001; Petri et al., 2013a), however, little information was available on the time course of hindgut microbial adaptation to HG diets using high-throughput technology. In the present study, we used MiSeq sequencing methods to assess the dynamic shifts in the microbial adaptation of sheep’s colon in response to an HG diet. In accordance with a previous study that reported feeding an HG diet can reduce the diversity and functionality of the microbiome in digestive tract of ruminant (Khafipour et al., 2016), our results showed a linear decrease in the bacterial richness and diversity of microbial communities in the hindgut with number of days fed an HG diet. In addition, PCoA and AMOVA results revealed significant difference in the composition and structure of colonic mucosa-associated bacterial communities among the four groups. These results suggest that colonic mucosa-associated microbiome were affected by dietary factors and may also require time to adapt to dietary changes, and 4 week is not sufficient to complete the microbiota adaptive response. A plausible explanation for the gradually adaptation in colonic bacterial community may be the higher and gradual changes in starch content (as well as other nutrients) in colonic digesta in comparison with the forage diet given to CON group. Gradual changes in the supply of starch and other nutrients entering colon after dietary change may lead to gradual changes in composition of colonic mucosa-associated microbiota. In addition, the increase in the concentration of VFA and a low pH in colonic digesta of HG groups may make a contribution to the shift in the mucosa-associated bacterial community in sheep.

In accordance with earlier reports that studied the hindgut bacterial communities of goats (Ye et al., 2016) and cows (Plaizier et al., 2016), the present study showed that members of the phylum Firmicutes were dominant in samples from colonic mucosa of sheep fed an HG diet, emphasizing their role in polysaccharide degradation. In contrast, Spirochaetae are the predominant bacterial phyla in colonic mucosa of the CON group. Spirochaetae has been reported as the natural intestinal flora in the gut mucosa of pre-weaned calves (Malmuthuge et al., 2014) and the caecal mucosa of goats (Liu et al., 2014). The high abundance of genus *Treponema*, which are revealed as cellulolytic bacteria in the rumen (Piknova et al., 2008), may explain the high amount of Spirochaetae in the CON group in the present study.

At the genus level, our data revealed that HG feeding increased the abundance of some bacteria in the colonic mucosa of sheep. Of these genera, *Prevotella* (Khafipour et al., 2009; Flint et al., 2012) and *Oscillibacter* (Walker et al., 2011), as archenteric bacteria playing a role in starch degradation and utilization have been reported, *Roseburia* spp. and *Bifidobacterium* spp. are also known for their amylolytic activity in the human colon (Ze et al., 2012). It has been reported that HG feeding increased the abundance of unclassified Lachnospiraceae and unclassified Erysipelotrichaceae in the gut of goats (Huo et al., 2014) and cattle (Kim et al., 2014). Thus, in the current study, the higher percentage of *Prevotella, Oscillibacter, Roseburia, Bifidobacterium,* unclassified Lachnospiraceae, and unclassified Erysipelotrichaceae in the colonic mucosa of sheep fed an HG diet may be explained by the increased starch content that reached the colonic lumen. It has been reported that some *Clostridium* spp., as opportunistic pathogens, are causative agents of intestinal enteric diseases in goats (Garcia et al., 2013). Moreover, *Clostridium* phylotypes could adversely affect the mucosal barrier (Kellermayer et al., 2011). Thus, the increase in the abundance of *Clostridium_sensu_stricto_1* in the present study implies that feeding an HG diet may have a detrimental effect on the colonic health of sheep. In addition, the relative abundance of *Faecalibacterium*, which was described to produce acetate as a fermentation product (Duncan et al., 2002), significantly increased in HG28 group in the present study, explaining the soaring acetate concentration in HG28 in the lumen of the colon. In contrast, a very large percentage of *Treponema* was detected in the CON group in the present study. The possible explain is that the pure hay diet fed to the CON group provided suitable nutrient conditions for *Treponema* to grow, while the shift of nutrient sources in the HG groups resulted in its decrease (Piknova et al., 2008). The present study also revealed that HG feeding has a dynamic effect on the composition of colonic mucosa-associated microbiota, such as *Helicobacter*. This shift may be attributed to the toxic effect of the linearly increased VFA concentrations in the HG groups. Actually, some previous studies have revealed that organic acids, such as acetate and lactate, can freely enter bacterial cells by diffusion across the bacterial membrane and dissociate inside the bacterial cell, which may reduce the intracellular pH and damage the internal cell (Harold and Levin, 1974; Boenigk et al., 1989; Russell, 1992).

### Temporal Dynamics in the Predicted Molecular Functions of Colonic Mucosa-Associated Bacterial Microbiota

Previous studies revealed that the gut bacteria made an important functional contribution in maintaining the health of the host (Lichtman et al., 2015; Ussar et al., 2016). In the present study, our PICRUSt results, which served to predict the potential functions of bacteria, revealed that the most abundant gene categories in the colonic-mucosa associated microbiome of sheep were associated with the function of membrane transport, carbohydrate metabolism, amino acid metabolism, replication and repair, translation, and energy metabolism, which is similar to the results of previous studies that were conducted in goats (Ye et al., 2016) and sheep (Liu et al., 2017). The high level of these functional pathways highlights the vital function of metabolism (carbohydrate metabolism, amino acid metabolism, and energy metabolism) in microbial survival (Lamendella et al., 2011).

With the exception of the numerous similarities in the abundance of functional pathways, 24 bacterial functions were affected by the HG feeding. In the shifted functional pathways of the colonic mucosa, the relative abundance of genes associated with carbohydrate metabolism, amino acid metabolism, and lipid metabolism was up-regulated by HG feeding, indicating that an HG diet enhanced the metabolic function of the bacterial community. The current study showed that the relative abundance of genes associated with environmental adaptation decreased after being fed an HG diet for 7 days and then recovered to the CON level with days fed an HG diet. Previous studies have reported that the complex microbiota of the hindgut is highly responsive to diet changes (Liu et al., 2014; Ye et al., 2016), thus the coordinated change in gene expression of environmental adaptation may suggest that the HG diet was stressful to colonic mucosal bacteria at the initial feeding stage, and that long-term exposure to HG diet can result in the adaption of the bacterial community to an HG diet. In addition, the number of genes associated with the immune system decreased after HG feeding, implying that long-term HG feeding may adversely affect host immune function. In summary, our data revealed that long-term HG feeding could change the function of the mucosal-associated bacteria in the colon of sheep.

### Temporal Dynamics in Colonic Mucosal Injury and the Correlation with Luminal pH and Mucosa-Associated Microbiota Abundance

The dynamic changes in colonic fermentation and the mucosal bacterial community may affect the histological integrity and function of the colonic mucosa, further inducing the local immune response of the host. In our research, an HG diet caused the slough of the surface layer epithelium, and the severity of the slough gradually increased with number of days fed an HG diet. The linear decrease of claudin-1 and the cubic decrease of claudin-4, which are conducive to mucosal barrier function in the large intestine (Poritz et al., 2011; Ye et al., 2016), implying that HG diet feeding may result in damage to barrier function. Several factors might account for the mucosal injury in the HG groups. Firstly, massive amounts of carbohydrates in the colon of sheep fed an HG diet could enhance the osmotic pressure of the digesta, and this enhanced pressure may damage the epithelial barrier and raise the permeability of the colonic mucosa (Gressley et al., 2011). Secondly, luminal pH plays an important role in maintaining the stable function of the epithelial barrier. Earlier studies revealed that lower GI pH may promote VFA diffusion across the mucosal cells and dissociation inside the cell, and significantly reduce the intracellular pH, eventually resulting in internal cell damage (Argenzio and Meuten, 1991; Andrews et al., 2005). Our results also showed that the concentration of total VFA linearly increased with number of days fed an HG diet, which may contribute to the colonic mucosal injury.

Consistent with the previous studies that HG feeding resulted in colon inflammation in goats (Liu et al., 2014; Ye et al., 2016), the current study showed that the expression of inflammation cytokines and TLRs, such as IL-1β, IL-6, TNF-α, and TLR3, increased linearly with days fed HG diet. Previous studies have reported the relationship between reduced pH and inflammation in the hindgut of goats (Liu et al., 2014; Ye et al., 2016), and our correlation analysis showed that the mRNA level of TNF-α was negatively related to pH, implying that linearly reduced pH may result in linearly increased cytokine expression with increasing number of days of being fed an HG diet. As mentioned earlier, lower pH may accelerate the entering of VFA to mucosal cells, acidify the cells and harmfully affect the Na^+^ transportation, eventually damaging the cells (Andrews et al., 2005). In addition to the consistently decreased pH, some potentially pathogenic intestinal bacteria can also induce inflammation (Liu et al., 2014; Ye et al., 2016). Our correlation results also showed that the mRNA expression of IL-1β and TNF-α were positively related to enrichments in *Clostridium_sensu_stricto_1* in the colon of sheep. As mentioned previously, some *Clostridium* spp. have a detrimental effect on mucosal health, thus the colonic epithelial inflammation may be partially caused by the enrichment of *Clostridium_sensu_stricto_1* in mucosa of sheep fed an HG diet.

## Conclusion

To the best of our knowledge, the present study is the first to survey the dynamic shifts of colonic mucosal microbiomes during HG feeding using high-throughput technology in sheep. Our data revealed that feeding an HG diet linearly reduced the diversity and richness of the colonic mucosa-associated microbiota, with significantly-dynamic shifts in the abundance of *Prevotella, Coprococcus, Roseburia*, *Treponema,* and *Helicobacter*. These results indicate that starch that flowed into the hindgut altered the microbial fermentation and the composition and potential function of the mucosa-associated bacterial communities in the colon, and resulted in high levels of VFA and a low pH in digesta of colon. In addition, feeding an HG diet linearly decreased the mRNA expression of claudin-1 and increased expression of IL-1β, IL-6, and TNF-α in the colonic mucosa as the duration of the HG feeding extended. Correlation analysis revealed that consistently reduced pH and a shift in mucosal bacteria community may contribute to local inflammation in colonic epithelium. In general, our data showed that HG feeding increased colonic fermentation and dynamically altered the composition and function of colonic mucosal bacterial communities, which eventually caused colonic mucosal damage and resulted in colonic dysfunction, and 4 weeks is not sufficient to complete the adaptive response to an HG diet.

## Author Contributions

YW carried out all the experiments and analyzed the data; LX participated in animal care; JL revised the manuscript; SM and WZ were responsible for study design; YW and SM were responsible for writing the manuscript.

## Conflict of Interest Statement

The authors declare that the research was conducted in the absence of any commercial or financial relationships that could be construed as a potential conflict of interest.
