# Supplementary material for: A High Grain Diet Dynamically Shifted the Composition of Mucosa-Associated Microbiota and Induced Mucosal Injuries in the Colon of Sheep
Source: Front Microbiol. 2017 Oct 26;8:2080. doi: 10.3389/fmicb.2017.02080 (PMC5662643; doi:10.3389/fmicb.2017.02080)
Supplement: Supplementary file 1 [file Data_Sheet_1.DOCX]

Table S1. Ingredient and chemical composition of the diet (dry matter basis).

| Item | Diet | |
| --- | --- | --- |
|  | Hay | High grain diet |
| Ingredient composition, % DM |  |  |
| Oat hay | 63.40 | 26.00 |
| Alfalfa hay | 33.00 | 14.00 |
| Corn meal | 0 | 34.20 |
| Wheat meal | 0 | 18.00 |
| Soybean meal | 0 | 4.20 |
| CaCO_3_ | 1.00 | 1.00 |
| NaCl, salt | 0.40 | 0.40 |
| CaHPO_4_ | 1.20 | 1.20 |
| Mineral and vitamin supplement^1^ | 1.00 | 1.00 |
| Nutrient composition^2^ |  |  |
| DE, MJ/kg DM | 8.88 | 11.73 |
| Crude protein, % DM | 11.18 | 11.92 |
| Crude fat, % DM | 2.09 | 2.49 |
| Crude fiber, % DM | 28.41 | 12.90 |
| Neutral detergent fiber,% DM | 44.45 | 24.54 |
| Acid detergent fiber, % DM | 19.52 | 10.15 |
| Crude ash, % DM | 8.34 | 4.53 |
| Starch, % DM | 3.25 | 32.34 |

^1^ Contained 16% calcium carbonate, 102 g/kg of Zn, 47 g/kg of Mn, 26 g/kg of Cu, 1,140 mg/kg of I, 500 mg/k of Se, 340 mg/kg of Co, 17,167,380 IU/kg of Vitamin A, 858,370 IU/kg of vitamin D, and 23,605 IU/kg of vitamin E.

^2^ Values were analyzed based on the Chinese Feed Database (2015).

Sheep assigned to CON (n = 5), HG7 (n = 5), HG14 (n = 5) and HG28 (n = 5) received a high grain diet for 0, 7, 14 and 28 days, respectively.

Table S2 Gene names, primer, sequences for real-time quantitative PCR analysis

| Gene Name | Primer sequence (5’-3’) | Source |
| --- | --- | --- |
| IL-1β | Forward: CATGTGTGCTGAAGGCTCTC | Liu et al., 2013 |
|  | Reverse: AGTGTCGGCGTATCACCTTT |  |
| IL-6 | Forward: CCAATCTGGGTTCAATCAGG | Liu et al., 2013 |
|  | Reverse: ACCCACTCGTTTGAGGACTG |  |
| IL-10 | Forward: TTAAGGGTTACCTGGGTTGC | Liu et al., 2013 |
|  | Reverse: CCCTCTCTTGGAGCATATTGA |  |
| TNF-α | Forward: CATGTGTGCTGAAGGCTCTC | Liu et al., 2013 |
|  | Reverse: AGTGTCGGCGTATCACCTTT |  |
| IFN-γ | Forward: TTAAGGGTTACCTGGGTTGC | Liu et al., 2013 |
|  | Reverse: CCCTCTCTTGGAGCATATTGA |  |
| TLR-3 | Forward: TCTTTTCGGGACTGTTGACC | Liu et al., 2015 |
|  | Reverse: AAATCCCCCATCCAAGGTAG |  |
| TLR-4 | Forward: GGTTTCCACAAAAGCCGTAA | Liu et al., 2015 |
|  | Reverse: AGGACGATGAAGATGATGCC |  |
| GAPDH | Forward: GGGTCATCATCTCTGCACCT | Wang et al. 2009 |
|  | Reverse: GGTCATAAGTCCCTCCACGA |  |

Table S3 Functional diversity of the colonic mucosa-associated microbiota of sheep. Sheep assigned to CON (n = 5), HG7 (n = 5), HG14 (n = 5) and HG28 (n = 5) received a high grain diet for 0, 7, 14 and 28 days, respectively.

| Item | Relative abundance (%) | | | | SEM | FDR |
| --- | --- | --- | --- | --- | --- | --- |
|  | CON | HG7 | HG14 | HG28 |  |  |
| Membrane Transport | 12.88 | 12.89 | 12.15 | 13.25 | 0.175 | 0.248 |
| Carbohydrate Metabolism | 9.35^c^ | 10.40^ab^ | 10.00^bc^ | 10.54^a^ | 0.121 | 0.03 |
| Amino Acid Metabolism | 9.05^b^ | 9.81^a^ | 9.88^a^ | 9.67^ab^ | 0.102 | 0.042 |
| Replication and Repair | 8.91 | 8.75 | 8.76 | 8.67 | 0.036 | 0.218 |
| Translation | 6.14^a^ | 5.58^b^ | 5.79^ab^ | 5.61^b^ | 0.064 | 0.03 |
| Energy Metabolism | 5.5 | 5.73 | 5.88 | 5.73 | 0.066 | 0.479 |
| Poorly Characterized | 4.97^a^ | 4.72^ab^ | 4.64^b^ | 4.67^b^ | 0.037 | 0.03 |
| Cell Motility | 4.35^a^ | 2.80^b^ | 3.33^ab^ | 2.99^b^ | 0.164 | 0.03 |
| Nucleotide Metabolism | 4.06^a^ | 3.93^b^ | 4.01^ab^ | 3.90^b^ | 0.021 | 0.033 |
| Metabolism of Cofactors and Vitamins | 3.94 | 4.27 | 4.36 | 4.24 | 0.057 | 0.231 |
| Cellular Processes and Signaling | 3.84^b^ | 4.08^a^ | 4.05^a^ | 4.03^a^ | 0.028 | 0.032 |
| Genetic Information Processing | 2.81^a^ | 2.65^cb^ | 2.75^ab^ | 2.61^c^ | 0.025 | 0.03 |
| Lipid Metabolism | 2.73^b^ | 2.87^a^ | 2.84^a^ | 2.86^a^ | 0.017 | 0.032 |
| Transcription | 2.63^b^ | 2.95^ab^ | 2.79^ab^ | 3.07^a^ | 0.055 | 0.03 |
| Folding, Sorting and Degradation | 2.60^a^ | 2.39^b^ | 2.50^ab^ | 2.33^b^ | 0.033 | 0.03 |
| Metabolism | 2.24 | 2.38 | 2.28 | 2.34 | 0.021 | 0.073 |
| Glycan Biosynthesis and Metabolism | 2.08 | 1.94 | 2.1 | 1.77 | 0.053 | 0.056 |
| Signal Transduction | 2.01^a^ | 1.71^b^ | 1.77^b^ | 1.73^b^ | 0.03 | 0.03 |
| Enzyme Families | 1.96^b^ | 2.12^a^ | 2.07^ab^ | 2.13^a^ | 0.018 | 0.03 |
| Metabolism of Terpenoids and Polyketides | 1.58^c^ | 1.64^a^ | 1.64^a^ | 1.59^bc^ | 0.009 | 0.03 |
| Xenobiotics Biodegradation and Metabolism | 1.52^b^ | 1.76^a^ | 1.69^a^ | 1.69^a^ | 0.028 | 0.032 |
| Metabolism of Other Amino Acids | 1.44 | 1.44 | 1.46 | 1.4 | 0.012 | 0.35 |
| Biosynthesis of Other Secondary Metabolites | 0.77 | 0.87 | 0.81 | 0.88 | 0.016 | 0.076 |
| Cell Growth and Death | 0.57 | 0.51 | 0.52 | 0.52 | 0.008 | 0.293 |
| Infectious Diseases | 0.38^a^ | 0.34^b^ | 0.37^ab^ | 0.34^b^ | 0.006 | 0.03 |
| Endocrine System | 0.36 | 0.29 | 0.27 | 0.29 | 0.013 | 0.293 |
| Transport and Catabolism | 0.27 | 0.24 | 0.22 | 0.23 | 0.007 | 0.123 |
| Environmental Adaptation | 0.22^a^ | 0.17^b^ | 0.19^a^ | 0.18^a^ | 0.006 | 0.03 |
| Neurodegenerative Diseases | 0.18^a^ | 0.14^ab^ | 0.22^a^ | 0.11^b^ | 0.018 | 0.045 |
| Signaling Molecules and Interaction | 0.13^b^ | 0.15^a^ | 0.15^a^ | 0.14^ab^ | 0.003 | 0.03 |
| Cancers | 0.12^a^ | 0.10^b^ | 0.11^ab^ | 0.10^b^ | 0.002 | 0.03 |
| Metabolic Diseases | 0.1 | 0.1 | 0.09 | 0.1 | 0.001 | 0.056 |
| Immune System | 0.10^a^ | 0.09^b^ | 0.09^ab^ | 0.09^b^ | 0.001 | 0.032 |
| Nervous System | 0.09 | 0.1 | 0.1 | 0.1 | 0.002 | 0.054 |
| Immune System Diseases | 0.04 | 0.04 | 0.04 | 0.04 | 0.001 | 0.932 |
| Digestive System | 0.03 | 0.03 | 0.02 | 0.03 | 0.001 | 0.518 |
| Excretory System | 0.03^a^ | 0.02^ab^ | 0.01^b^ | 0.01^b^ | 0.002 | 0.03 |
| Circulatory System | 0.03^a^ | 0.01^ab^ | 0.03^a^ | 0.01^b^ | 0.005 | 0.032 |
| Cardiovascular Diseases | <0.01^b^ | <0.01^b^ | <0.01^b^ | <0.01^a^ | <0.001 | 0.03 |
| Cell Communication | 0 | 0 | 0 | <0.01 | <0.001 | 0.424 |

Figure S1 The rarefaction curves of the colonic mucosa-associated microbiota of hay-fed (CON) and concentrate-fed sheep (HG7–28). Sheep assigned to CON (n = 5), HG7 (n = 5), HG14 (n = 5) and HG28 (n = 5) received a high grain diet for 0, 7, 14 and 28 days, respectively.





Figure S2 The number of OTUs shared among each stage of the adaptation programs in colonic mucosa of hay-fed (CON) and high grain diet-fed sheep (HG7–28). Sheep assigned to CON (n = 5), HG7 (n = 5), HG14 (n = 5) and HG28 (n = 5) received a high grain diet for 0, 7, 14 and 28 days, respectively.


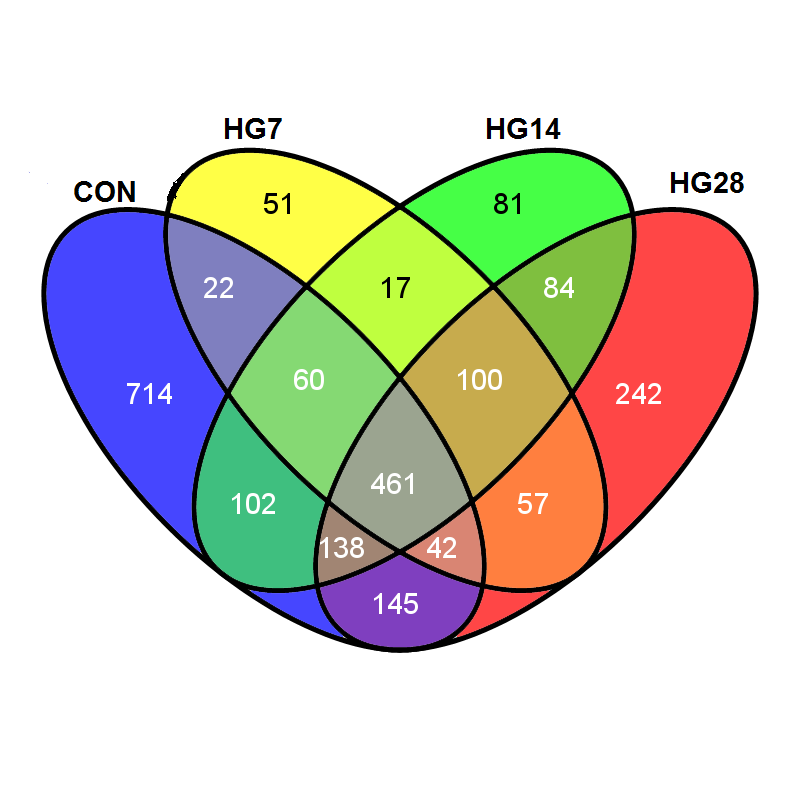


Figure S3 The average relative abundance of phylum (% of total sequences) in colonic mucosa.


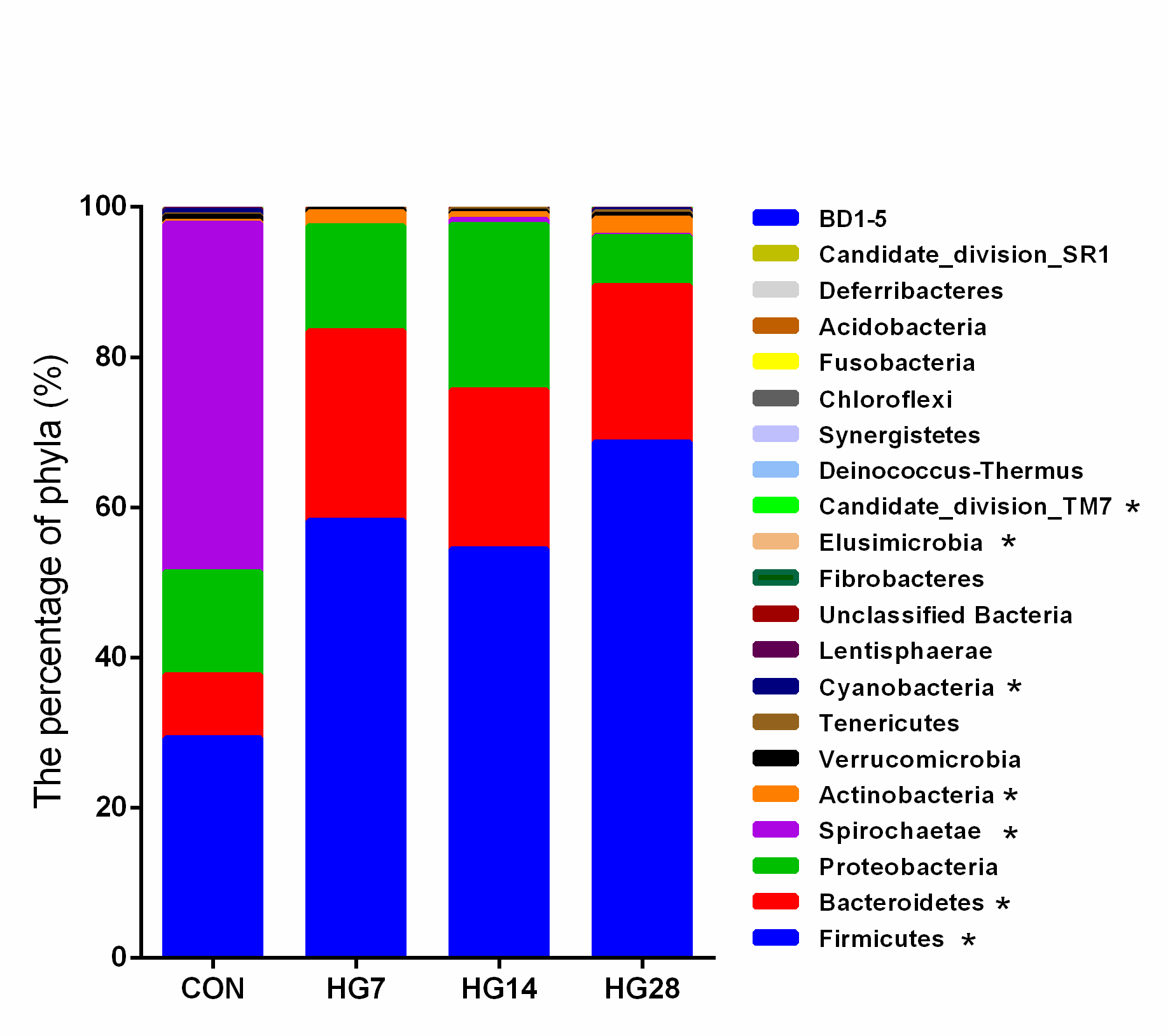


Figure S4 The average relative abundance of genus (% of total sequences) in colonic mucosa. Only genera with a relative abundance ≥ 1% in at least one treatment were showed.
